# Unraveling the diversification history of grasshoppers belonging to the “*Trimerotropis pallidipennis”* (Oedipodinae: Acrididae) species group: a hotspot of biodiversity in the Central Andes

**DOI:** 10.7717/peerj.3835

**Published:** 2017-09-29

**Authors:** Noelia Verónica Guzmán, Silvia Mónica Pietrokovsky, Maria Marta Cigliano, Viviana Andrea Confalonieri

**Affiliations:** 1 Facultad de Ciencias Exactas y Naturales, Universidad de Buenos Aires y Consejo Nacional de Investigaciones Científicas y Tecnológicas, Instituto de Ecología, Genética y Evolución (IEGEBA), Buenos Aires, Argentina; 2 Consejo Nacional de Investigaciones Científicas y Tecnológicas, Museo de La Plata, Universidad Nacional de la Plata, Centro de Estudios Parasitológicos y de Vectores (CEPAVE), La Plata, Buenos Aires, Argentina

**Keywords:** Species delimitation, Grasshopper, Biogeography, Phylogenetic

## Abstract

The Andean Mountain range has been recognized as one of the biodiversity hotspots of the world. The proposed mechanisms for such species diversification, among others, are due to the elevation processes occurring during the Miocene and the intensive glacial action during the Pleistocene. In this study we investigated the diversification history of the grasshopper *Trimerotropis pallidipennis* species complex which shows a particularly wide latitudinal and altitudinal distribution range across the northern, central and southern Andes in South America. Many genetic lineages of this complex have been so far discovered, making it an excellent model to investigate the role of the central Andes Mountains together with climatic fluctuations as drivers of speciation. Phylogenetics, biogeographic and molecular clock analyses using a multi-locus dataset revealed that in Peru there are at least two, and possibly four genetic lineages. Two different stocks originated from a common ancestor from North/Central America—would have dispersed toward southern latitudes favored by the closure of the Panama Isthmus giving rise to two lineages, the coastal and mountain lineages, which still coexist in Peru (i.e., *T. pallidipennis* and *T. andeana*). Subsequent vicariant and dispersal events continued the differentiation process, giving rise to three to six genetic lineages (i.e., clades) detected in this study, which were geographically restricted to locations dispersed over the central Andes Mountains in South America. Our results provide another interesting example of “island diversification” motored by the topography plus unstable climatic conditions during the Pleistocene, pointing out the presence of a hotspot of diversification in the Andean region of Peru.

## Introduction

The Neotropical Biogeographic Region is characterized by a remarkable biodiversity (Myers et al., 2000), but the origin of species richness remains controversial. The formation of the Andes might have favored diversification, creating biodiversity hotspots (especially in the tropics) that contain 6.7% and 5.7% of all endemic plants and vertebrates in the world, respectively (Myers et al., 2000; Morawetz & Raedig, 2007). The high habitat diversity in the Andes, which most probably resulted from differences in orogeny, topography, soils, climate and elevation, may have played an important role in biodiversity (Pocco et al., 2013).

**Figure 1:**
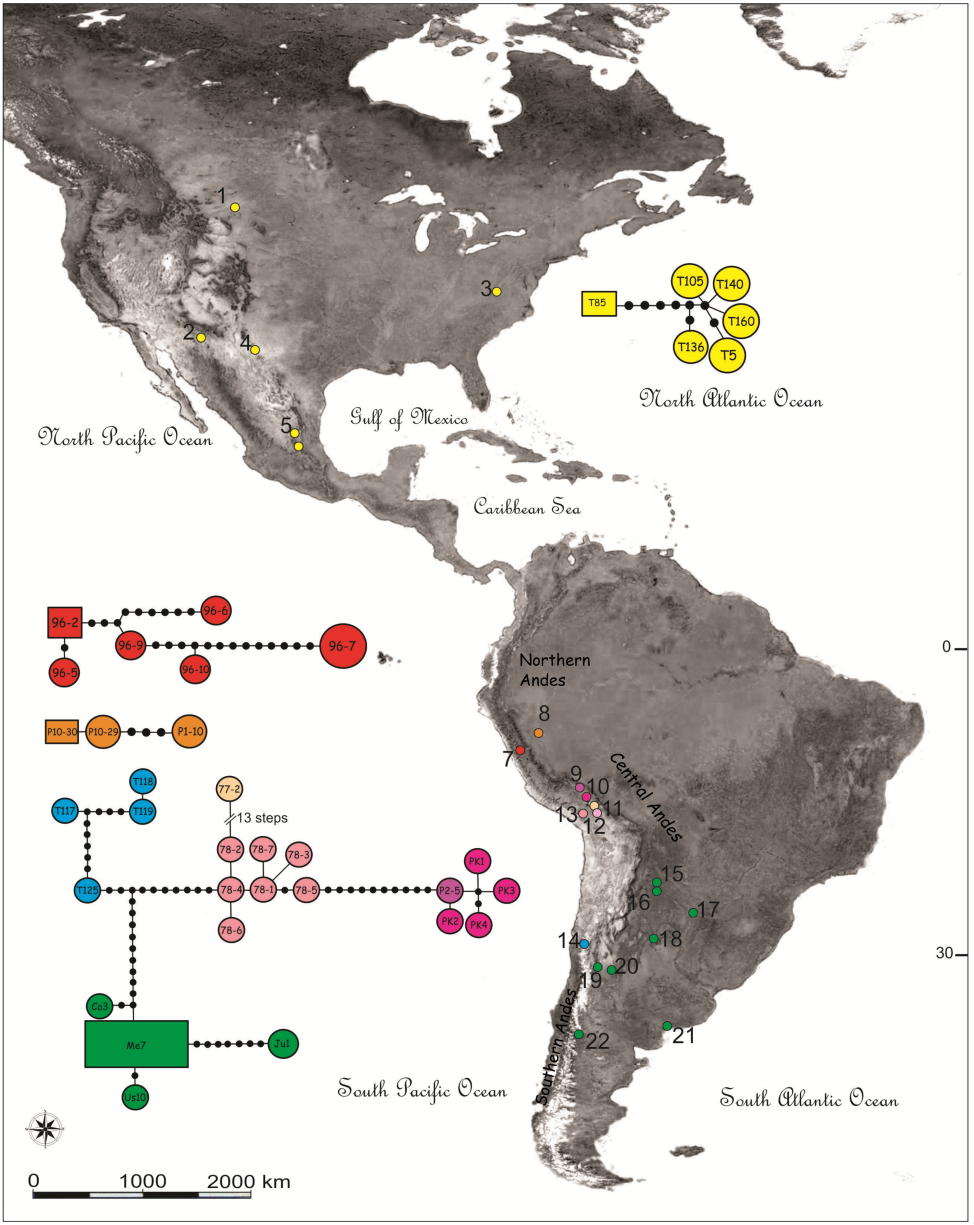
Map showing the sampling locations and haplotype networks built with a concatenated data set of COI and NADH5 mitochondrial genes of species of the *Trimerotropis pallidipennis* complex. Map showing the sampling locations of species of the *Trimerotropis pallidipennis* complex in North-Central and South America and haplotype networks built with a concatenated data set of COI and NADH5 mitochondrial genes. Haplotypes composed of a single specimen receive its ID. Haplotypes with many specimens are called after one of them, as follows: haplotype Me7 comprises Vi1, Cha3, C12C, SA3, G2; haplotype 96-2, 96-8; haplotype 96-7,96-4, 96-1; haplotype Ju1, Ju2; haplotypes T119, T116; haplotypes T126, T125; haplotype P10-30, P10-28. For location ID see Table S1. Different colors in Peru denote different localities/haplogroups; for color ID in other regions, see Fig. 2.

**Figure 2:**
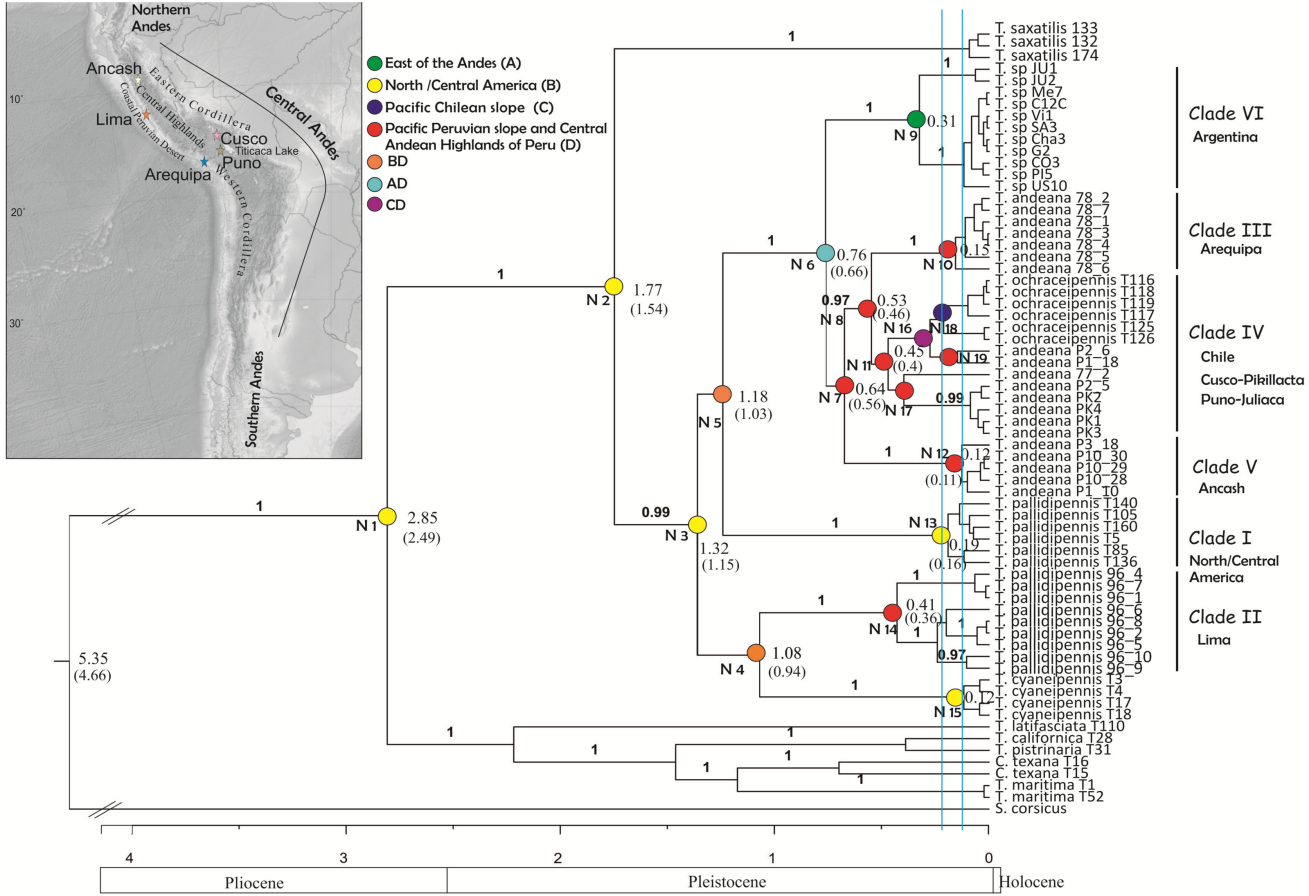
Tree topology obtained from the partitioned Bayesian analysis of the four gene fragments COI, NADH5, ITS2 and HIS3 dataset for the *Trimerotropis pallidipennis* species complex. Tree topology obtained from the partitioned Bayesian analysis of the four gene fragments COI, NADH5, ITS2 and HIS3 dataset for the *Trimerotropis pallidipennis* species complex from North and South America and related species; *T.* = *Trimerotropis*; *S.* = *Sphingonotus*; C. = Conozoa. Ancestral area optimization obtained from the program RASP is indicated. Numbers above branches indicate posterior probabilities. Numbers in each node indicate its age. The node age was calculated according to Allegrucci, Trucchi & Sbordoni (2011) and Papadopoulou, Anastasiou & Vogler (2010) (Node ages outside and within brackets, respectively). Individual IDs correspond to those in Table S1. Blue lines indicate the two maximum-likelihood transition point of the switch in branching rates from interspecific to intraspecific events (approximately 220,000 and 125,000 years ago) estimated by the GMYC. Major geological epochs are given.

Three subdivisions have been identified in the Andes (Gansser, 1973): southern Andes of Argentina and Chile, central Andes of Bolivia and Peru and northern Andes of Ecuador, Colombia and Venezuela (Figs. 1 and 2). The central Andes is the longest subdivision (4,000 km), encompasses the most mountainous areas and includes the geomorphologic unit of the Central Highlands (about 4,000 m of elevation), which is bounded by the Western and Eastern Cordilleras with peaks of more than 6 km (Garzione et al., 2008; Gonzalez & Pfiffner, 2011). The Central Highlands unit widens into the Altiplano to the southeast, and these, together with the Eastern Cordillera, may have reached their current elevation at about 10 million years ago (Mya) (Gregory-Wodzicki, 2010).

During the Pleistocene, the Peruvian mountains in central Andes showed a complex landscape varying from the eastern wet to the western dry slopes, which included landscape features such as glaciated peaks, dry valleys, and humidity gradients (Graham, 2009; Klein, Seltzer & Isacks, 1999). High altitudes together with globally low temperatures at about 3–5 Mya might have led to the rapid island-like radiation of high-elevation species (Graham, 2009; Young et al., 2002; Turchetto-Zolet et al., 2013). Indeed, numerous phylogeographic studies on co-distributed taxa demonstrate that mountain systems like the Southern Alps in New Zealand, the Himalayas, the Pyrénées and the Andes, could have played an important role in the generation of new lineages during the glacial periods of the Pleistocene (Sérsic et al., 2011; Wallis et al., 2016).

Particularly in Peru, there are many endemic montane species belonging to *Ilex* (Aquifoliaceae), *Brunellia* (Brunelliaceae), *Peperomia* and *Piper* (Piperaceae), *Styrax* (Stryacaceae), *Symplocos* (Symplocaceae) and the genus *Arnaldoa* (Asteraceae); the latter is represented by four species, most of which are mainly restricted to xerophytic environments in inter-montane areas (Young & León, 2001). In amphibians, the genus *Telmatobius* depicts a case of endemism (Saez et al., 2014). In birds, the species of the genus *Muscisaxicola*, a primarily Andean group of tyrant-flycatchers, appear to have diversified during the Middle and Late Pleistocene (Chesser, 2000). Among insects, several grasshopper species show signs of recent diversification in the North and Central Andes (Cigliano & Amedegnato, 2010; Cigliano et al., 2010; Cigliano, Pocco & Lange, 2011; Pocco et al., 2013; Pocco et al., 2015). The *Trimerotropis pallidipennis* species complex is a group of amphitropical band-winged grasshoppers widely distributed in arid and semiarid regions of the Americas, from southwestern Canada to southern Argentina (Otte, 1984; Husemann et al., 2013). This broad distribution may be due to their high flight capacity and nocturnal dispersal activity (Otte, 1984). It belongs to the subfamily Oedipodinae, which together with others Neotropical Acridoidea like Acridinae and Gomphocerinae (Acrididae), possibly constitute one of the last strata of the Acridoidea fauna entering into South America from the Nearctic region during the Pliocene. The Andes mountain range could have served as the main route of dispersion for these grasshoppers (Carbonell, 1977).

In North-Central America the complex is distributed throughout western USA and extends southward to arid and semi-arid areas of Mexico up to the state of Oaxaca, where it is represented by a single species, *Trimerotropis pallidipennis* (Husemann et al., 2013). It is absent southward until conditions become favorable in the southern Andes of Ecuador and Peru; it has a continuous distribution from the Peruvian Pacific slope to the south through Bolivia, Chile and Argentina, living at altitudes ranging from sea level (e.g., Lima and Chosica in Peru and Buenos Aires in Argentina) to as high as 3,000–4,000 m.a.s.l. (e.g., Ancash and Matucana in Peru and Jujuy in Argentina). Rehn (1939) described the subspecies *T. pallidipennis andeana* from the Peruvian Puna areas at altitudes from 3,340 to 4,000 m.a.s.l. This author emphasized that *T. pallidipennis andeana* is the high-altitude representative of *T. pallidipennis,* at least in part of the Peruvian Puna, and that the morphological characteristics useful in identifying the subspecies may become less evident southward. Moreover, Rehn (1939) proposed that Peruvian populations could have originated from different “stocks”, considering that specimens from the Peruvian Pacific slope (*T. pallidipennis pallidipennis*) and the Puna areas were differentiated only by subtle morphological variations. However, in his catalogue of the North American grasshoppers, Otte (1995) listed *T. pallidipennis andeana* at the species status. Other five species have been described for the complex in South America, all of them being distributed in Chile: *T. ochraceipennis* (Blanchard, 1851), *T. atacamensis* (Philippi), *T. flavipennis* (Philippi), *T. chloris* (Philippi) and *T. irrorata* (Philippi). The latter four nominal species probably synonyms of *T. ochraceipennis* according to Amedegnato & Carbonell (2001). More recently, Husemann et al. (2013) performed a species delimitation analysis based on molecular markers revealing that *T. pallidipennis* species complex may be composed of at least three distinct genetic lineages: *Trimerotropis pallidipennis* from North America, *Trimerotropis ochraceipennis* from Chile and a *Trimerotropis* species from Argentina (hereafter T. sp.). Interestingly, these authors pointed that the group of Peruvian specimens from the Andean Puna of Cusco and Ancash—taxonomically assigned to *T. andeana*—was apparently composed of an admixture of two or more distinct biological units, and further proposed that these lineages could be undergoing a differentiation process. Indeed, a recent survey of grasshoppers in this area revealed high levels of endemism and numerous species new to science (Cigliano, Pocco & Lange, 2011; Pocco et al., 2013; Pocco et al., 2015).

Considering that the *Trimerotropis pallidipennis* species complex shows a particularly wide latitudinal and altitudinal distribution range across the northern, central and southern Andes in South America, and that many genetic lineages of this complex have been so far discovered, it constitutes an excellent model to investigate the role of the central Andes together with climatic fluctuations as drivers of speciation, compared to other regions in South America. For this purpose, in the present study we included new Peruvian specimens from the Central Andean Highlands and the Pacific Desert slope, and analyzed this enlarged sample using different phylogeographic and phylogenetic approaches. In addition, we performed a new biogeographic analysis to reconstruct the diversification history of the *Trimerotropis* complex across the Central Andes.

## Materials and Methods

### Sample collection

We included in the analysis 52 adults of the *Trimerotropis* species complex from 26 localities from USA, Mexico, Peru, Chile and Argentina, along the Andes (Table S1). Twenty-two of these specimens collected from five localities in Peru during summer 2013 were analyzed for the first time in this study. Hind legs were preserved in 100 % ethanol until DNA extraction. The following species were included as outgroups: *Sphingonotus corsicus* (*n* = 1), *Conozoa texana* (*n* = 2), *Trimerotropis pristinaria* (*n* = 1), *T. latifasciata* (*n* = 1), *T. cyaneipennis* (*n* = 4), *T. californica* (*n* = 1), *T. maritima* (*n* = 2) and *T. saxatilis* (*n* = 3). (Fig. 1, see Table S1 for specimens used and associated Genbank accession numbers).

### DNA extraction, PCR amplification and sequencing

Total genomic DNA was extracted from tissue of the hind leg that had been preserved in ethanol using the DNeasy Tissue Kit (Qiagen, Valencia, CA, USA). We used a polymerase chain reaction (PCR) to amplify two mitochondrial (mtDNA) and two nuclear (nDNA) DNA sequences: (1) the mitochondrial dehydrogenase subunit 5 gene fragment (NADH5), (2) the mitochondrial cytochrome c oxidase subunit I gene fragment (COI), (3) the internal transcribed spacer 2 (ITS2); and (4) the histone 3 gene (HIS3) fragment. Detailed information on primers can be found in Husemann et al. (2013). DNA fragments were amplified in 50-uL reactions consisting of 1× reaction buffer, 3 mM MgCl_2_, 1 unit of *Taq-* DNA-Polymerase (Invitrogen, Argentina), 0.1 mM of each dNTP, 100 ng of each primer (Invitrogen, Argentina) and 50–100 ng of DNA template. Polymerase chain reactions were run under the following conditions: 94 °C for 3 min, followed by 30 cycles of 94 °C for 1 min (denaturation), 48–60 °C (NAHD5 50 °C, COI 46 °C, HIS3 57 °C, ITS2 55 °C) for 1 min (annealing) and 72 °C for 2 min (elongation), with a final elongation step at 72 °C for 10 min. PCR Products were purified with a Purification Kit (AccuPrep; Bioneer Corporation, Daejeon, South Korea) to eliminate unused reagents. Finally, these were sequenced at the “Unidad de Secuenciación y Genotipificado” (FCEyN; UBA, Buenos Aires, Argentina). Sequences were inspected, trimmed and aligned using Geneious v.7.0.6.

Information on levels of sequence variation was obtained through MEGA v.7.0.21 program (Koichiro et al., 2013).

### Phylogenetic analysis

A phylogeny was constructed in a Bayesian framework for all four gene fragments of 67 individuals (52 specimens belonging to the ingroup and 15 to the different outgroups (Table S1), which were simultaneously analyzed using the program BEAST v.1.8.2 (Drummond et al., 2012); a Markov chain Monte Carlo (MCMC) simulation was run for 10 million generations, sampling trees every 1,000 generations. Each genetic region was treated as unlinked for substitution models but as linked for trees. The optimal evolutionary model for each dataset was inferred using JModelTest (Posada, 2008), on the basis of the Akaike Information Criterion (AIC) (Akaike, 1973, 1974), as suggested by Posada & Buckley (2004). Tracer v.1.6 was used to verify proper “mixing” of chains. TreeAnnotator v.1.7.5 (Drummond et al., 2012) was used to choose the maximum clade credibility tree with the “mean node heights” option from the output trees. Output parameters were examined in Tracer v.1.5 to determine whether the effective sample size of the parameters was >200. The ages of nodes were determined assuming a COI substitution rate of 3.54% and 3.2% pairwise divergence per million years (Myr) (Papadopoulou, Anastasiou & Vogler, 2010; Allegrucci, Trucchi & Sbordoni, 2011, respectively). The tree was also estimated using the “Metropolis-coupled Markov chain Monte Carlo” (MCMCMC) algorithm implemented in MrBayes v.3.2 (Huelsenbeck & Ronquist, 2001) with the concatenate dataset. Default prior distributions were used. The analysis was run over 1,000,000 generations with sampling every 100 generations. The tree space was explored by four chains (one cold chain and three incrementally heated chains). Convergence was checked with Tracer v.1.6 (Rambaut et al., 2014) and the average standard deviations of the split frequencies of the two runs were below 0.01.

A haplotype network was built with a concatenated data set that included both mitochondrial genes, using TCS program v.1.21 (Clement, Posada & Crandall, 2000), based on the statistical parsimony method described by Templeton, Crandall & Sing (1992).

Pairwise genetic distances between clades identified in the phylogenetic analysis were estimated using MEGA v.7.0.21 program (Koichiro et al., 2013).

### Assessment of genetic lineages

To infer the number of different genetic lineages, we applied the single- and multiple- threshold General Mixed Yule Coalescent (GMYC) (Pons et al., 2006; Fontaneto et al., 2007) methods using an ultrametric tree obtained from concatenated sequences of COI and NADH5 after removing identical haplotypes. GMYC aims to infer “species boundaries” based on the difference between the rate of coalescence within species and the rate of cladogenesis (Reid & Carsens, 2012). A likelihood ratio test (LRT) was applied to compare the likelihoods between the null model (i.e., entire sample belongs to a single species) and the alternative model (i.e., separate coalescent groups are nested within the species tree). Confidence limits correspond to threshold values ± 2 log L units around the ML (Maximum Likelihood) estimate. The ultrametric tree to be used as input was generated with BEAST v. 1.6.1 (Drummond & Rambaut, 2007) under strict molecular clock and applying a coalescent constant size as tree prior. We run a Markov chain Monte Carlo with 10 million generations. To perform the GMYC analysis we used SPLITS package in R (http://r-forge.rproject.org/projects/splits/).

### Area reconstruction

A biogeographic analysis was carried out to reconstruct the distribution history of the *Trimerotropis pallidipennis* complex. We used three reconstruction approaches, which differ slightly in the allowed cladogenetic events (Buerki et al., 2011; Ronquist & Sanmartin, 2011): a statistical dispersal-vicariance analysis (S-DIVA; Yu, Harris & He, 2010) based on the original formulation of Ronquist (1997); a dispersal-extinction-cladogenesis analysis (Lagrange DEC model; Ree & Smith, 2008); and a statistical dispersal-extinction-cladogenesis analysis (Lagrange S-DEC model; Beaulieu, Tank & Donoghue, 2013) using the program RASP v. 3.2 (Yu et al., 2015). The analysis was based on 1,000 post-burn-in trees from the MrBayes analysis (see item “Phylogenetics analyses” in Material and Methods). *Trimerotropis* populations were clustered into four regions based on their geographic distribution: (A) East of the Andes, (B) North and Central America, (C) Chilean Pacific slope and (D) Peruvian Pacific slope and Central Andean Highlands of Peru.

## Results

### Molecular data and models

We amplified and sequenced four marker fragments (two mtDNA with a total of 1,642 base pairs and two nDNA with a total of 641 base pairs) (Table S1). ITS2 alignments required the addition of gaps because of the presence of indels. No indels were incorporated in the COI, NADH5 and HIS3 datasets. The base frequencies of the four markers are typical for insects (Lin & Danforth, 2004). Both mitochondrial markers had more parsimony-informative sites (COI: 86/646 and NADH5: 116/996) than the nuclear ones (HIS3: 2/315 and ITS2: 1/327), indicating that they are better for distinguishing species and subspecies. Substitution rates/lineage/Myr inferred by BEAST were also higher for mitochondrial than for nuclear genes. The appropriate model of sequence evolution for each gene fragment obtained from the JModeltest program was: for COI, the general time reversible model (GTR) plus a rate variation among sites that follows a gamma distribution (G); for NADH5, the Hasegawa-Kishino-Yano model (HKW) with invariable sites (I), for ITS2, the HKW+G; and for HIS3, HKW.

### Phylogenetic and haplotype network analysis

Phylogenetic analysis using BEAST revealed two main groups (Fig. 2), one composed of *Trimerotropis* and *Conozoa* species from North America (*C. texana*, *T. maritima*, *T. californica, T. latifasciata* and *T. pistrinaria*) and the other of *Trimerotropis* species from North and South America (*T. saxatilis, T. cyaneipennis* and species of the *T. pallidipennis* complex). Within the latter group, *T. saxatilis* emerges basal to a clade containing (with a high Bayesian Posterior Probability (BPP) value, BPP = 1) all the individuals of the *T.pallidipennis* species complex and *T. cyaneipennis*. This clade splits into two groups: (i) a monophyletic group including all the individuals of *T. cyaneipennis* that resolves as sister lineage of all individuals of *T. pallidipennis* from the Peruvian Pacific slope (hereafter called Clade II, Fig. 2), and (ii) the remaining individuals of the *T. pallidipennis* species complex. Within the latter group (ii), the following lineages are successively resolved: a clade formed by all specimens from North and Central America (Clade I, Fig. 2); a lineage formed by all specimens from Argentina (Clade VI; Fig. 2); a lineage containing individuals from the geographic region of Ancash (Clade V, Fig. 2); and finally, a sister group comprising individuals of *T. andeana* from Arequipa (Clade III, Fig. 2) and individuals of *T. andeana* from Cusco, Pikillacta, Puno and Juliaca from Peru + *T. ochraceipennis* from Chile (Clade IV, Fig. 2). Therefore, all individuals of *T. andeana* would form a paraphyletic group because it includes specimens of *T. ochraceipennis* (Clades III, IV and V, Fig. 2). All lineages present high BPP values, except for nodes 4 and 5 (Fig. 2). Moreover, the analysis made with MrBayes based on the same concatenated matrix shows similar relationships as BEAST, except for a basal trichotomy formed by (*T. cyaneipennis* + Clade II), (Clade I) and (Clade III + Clade IV + Clade V + Clade VI) (figure not shown).

Haplotype networks were built including all individuals belonging to the *T. pallidipennis* species complex and *T. cyaneipennis* (Fig. 1). A total of five networks were obtained, each one including all haplotypes from: (i) *T. cyaneipennis* (not shown in Fig. 1); (ii) Clade I (*T. pallidipennis* from North America, Figs. 1 and 2); (iii) Clade II (*T. pallidipennis* from the Peruvian Pacific slope, Figs. 1 and 2); (iv) Clade V (*T. andeana* from Ancash, Figs. 1 and 2); and finally (v) Clade III + Clade IV + Clade VI (*T. andeana* from Arequipa, Cusco, Pikillacta, Puno and Juliaca; *T. ochraceipennis* from Chile and *T.* sp. from Argentina). The parsimony steps of the evolutionary distances among these networks exceed the number of steps required to be joined together.

These phylogenetic and network analyses clearly show that some individuals from Peru and Chile are more related among themselves, even included into the same genetic haplogroup, than to other Peruvian individuals from closer localities. It is also evident that in Peru there are at least three (Arequipa, Ancash, and Lima, Figs. 1 and 2) geographically constrained monophyletic lineages, which are supported by very high BPP values (Fig. 2) and separated by many (more than ten) parsimony steps (Fig. 1). Indeed, these levels of differentiation are reflected by the mean pairwise genetic distances estimated for the different group comparisons (Table 1): Clade I from North-Central America is the most differentiated group (0.034 ± 0.008), followed by Clade V from Ancash (0.024 ± 0.013), Clade II from Lima (0.016 ± 0.009), Clade IV from Chile-Cusco-Pikillacta-Puno-Juliaca (0.015 ± 0.012), Clade III from Arequipa (0.012 ± 0.012) and finally, Clade VI from Argentina (0.012 ± 0.011).

**Table 1:** Pairwise genetic distances (mean number of nucleotide substitutions per site for mitochondrial genes (COI-NADH5)) among groups (i.e., clades) of the *Trimerotropis pallidipennis* complex identified in Fig. 2.

**Pairwise genetic distances**						
	Clade I	Clade IV	Clade V	Clade VI	Clade III	Clade II
Clade I	–					
Clade IV	0.033	–				
Clade V	0.047	0.020	–			
Clade VI	0.030	0.006	0.016	–		
Clade III	0.031	0.005	0.015	0.001	–	
CladeII	0.028	0.013	0.024	0.008	0.008	–

### Assessment of genetic lineages

We applied the General Mixed Yule-coalescent (GMYC) method based on an ultrametric tree reconstructed from both concatenated mitochondrial genes. Only the “multiple” method gave significant results. The likelihood of the null model was 499.73; the ML of the GMYC model was 503.52; and the value of the LTR was 0.023 (i.e., significant). Two threshold times (approximately 220,000 and 125,000 years ago, Fig. 2) were found, indicating significant shifts in the rates of branching from coalescence to cladogenesis. These shifts apparently define 17 ML clusters (confidence interval: 5–17), or 32 ML entities (including cluster species plus single-specimen species; confidence interval: 8–36). According to this analysis, all monophyletic groups on the right side of the first threshold line can be considered as separate ML entities. For instance, Peruvian specimens could be separated into 9 ML entities: three for Clade II (Lima, Peru); one for Clade III (Arequipa, Peru); five for Clade IV, including *T. ochraceipennis*, (Cusco, Peru and Chile); and finally one for Clade V (Ancash, Peru). Clade VI (Argentina) can be split into two ML entities, and Clade I (North America) includes a single entity.

### Historical biogeography and divergence times

All S-DIVA, DEC and S-DEC analyses recovered the same areas for all nodes except for node 2, whose ancestor would have dispersed earlier to southern latitudes according to the DEC and S-DEC analyses (see Table S2).

Coincidentally, all analyses showed that the ancestor of the *Trimerotropis pallidipennis* species complex and *T. cyaneipennis* was distributed in North/Central America (Node 2 and 3, Fig. 2 and Table S2). The ancestor in Node 3 most probably separated into two stocks that extended their geographic ranges into Peru during Middle Pleistocene: one stock might have reached southern latitudes through the highlands of the Central Andes (i.e., Ancash, Arequipa, Cusco, Puno) (Node 3–5, Fig. 2), (see Table S2 for possible routes), while the other one might have spread until reaching the Peruvian coast (Lima), giving rise to Node 4. The ancestor in Node 4 also suffered a vicariant event that separated the Peruvian (Clade II) from the North American lineage (*T. cyaneipennis*). On the other hand, the ancestor in Node 5 extended its distribution southward (i.e., Argentina); subsequently, a vicariant event (Table S2), separated the North/Central American group (Clade I, Node 13) from the South American lineage (Node 6). Three vicariant events occurred at some time during the Middle-Late Pleistocene: one event separated the Argentinian populations (Node 9) from the Peruvian and Chilean populations (Node 7), and two events separated Clade III (from Arequipa, Peru, Node 10) and Clade V (from Ancash, Peru, Node 12) from the rest of the Peruvian and Chilean samples (Table S2).

## Discussion

### A new scenario of diversification for *Trimerotropis* species along the Andes

All the analyses performed here are consistent with the hypothesis that the *T. pallidipennis* species complex is probably composed of more than one genetic lineage: the phylogenetic analysis based on all four nuclear and mitochondrial genes shows six monophyletic clades (Clades I–VI) with high support value (except for Clade IV) and the GMYC analysis found 12 ML entities (excluding the outgroup species), of which nine lineages correspond to Peru, and the rest to Argentina and North/Central America.

On the basis of several morphological traits from the pronotum, caudal femora and wing-band coloration pattern, Rehn (1939) identified three South American “forms” of *Trimetrotropis* from Peru, Bolivia and Argentina: *T. p. pallidipennis*, *T. p. andeana*, and an atypical *T. p. pallidipennis*. Rehn (1939) found *T. p. pallidipennis* on the Peruvian Pacific slope, *T. p. andeana* at relatively high elevations in Peru, and *T. p. pallidipennis* in Bolivia and Argentina. However, we found no morphological diagnostic characters leading to a clear delimitation of these taxa, despite the large number of specimens from Peru, Bolivia and Argentina examined. Although we did find some differences in the characters described by him to distinguish *T. p. andeana*, variability increased as more specimens from the different regions of Peru were examined.. Notwithstanding this, our results partially agree with Rehn’s (1939) hypothesis because the Peruvian material examined in this study comprises at least two genetic lineages; one of these (called *T. p. pallidipennis* by Rehn (1939)), includes individuals from the Pacific desert coast of Peru (Clade II, Lima) and is more related to the North American clade and to *T. cyaneipennis* than to the other Peruvian specimens; the other lineage, *T. andeana* (called *T*. *p. andeana* by Rehn (1939) is represented by specimens from high altitudes in the Central Andes. Moreover, the “atypical specimens” of *T. p. pallidipennis* (Rehn, 1939) would correspond to individuals previously identified by Husemann et al. (2013) as *T.* sp. from Argentina. However, can the different lineages of *T. andeana* be regarded as different species? Although the large number of Peruvian species delimited by GMYC (i.e., nine) cannot be considered, *per se*, evidence enough for evaluating their specific status without additional data (Talavera, Dinca & Vila, 2013), we can anyway conclude that in Peru there are at least two (i.e., *T. pallidipennis* and *T. andeana*) and possibly four genetic lineages. Within *T. andeana* we found three monophyletic clades geographically restricted to locations dispersed over the particular Andean landscape, like Ancash (locality 8, Fig. 1) and Arequipa (locality 12, Fig. 1) in Peru (Clades III and V, respectively; Fig. 2), which are separated by several parsimony steps and relatively high mean nucleotide distances (Fig. 1, Table 1). Moreover, contrary to expectations, individuals of *T. andeana* collected from localities separated by only ca. 300 km, such as Puno, Juliaca and Arequipa (localities 11, 12 and 13, Fig. 1), show an evolutionary distance of 13 parsimony steps, while most individuals of *T.* sp. from Argentina, which are separated by thousands of kilometers show distances among haplotypes varying from 0 to 3 parsimony steps (Fig. 1), except for those in the northern locality of Jujuy. It is evident that this species complex underwent a diversification process triggered by Pleistocene climatic oscillation in the Central Andes, as it is also the case for other Acrididae species (Cigliano & Amedegnato, 2010; Pocco et al., 2013; Pocco et al., 2015), as well as for other taxa (Chesser, 2000; Hughes & Eastwood, 2006; Koscinski et al., 2008; Saez et al., 2014; Young et al., 2002).

On the other hand, the status of *T. ochraceipennis* should be revisited: first, because *T. andeana* resolves as paraphyletic unless *T. ochraceipennis* specimens are included in this group (Fig. 2); second, because specimens of *T. ochraceipennis* form a monophyletic clade, but with a low support value. It should be noted that the characters used by Rehn (1930) to distinguish *T. pallidipennis* from *T. ochraceipennis* (median carina of prozona with individual crested lobes and metazonal disk of pronotum with lateral carinated shoulders) were highly variable, and did not help to clearly differentiate between *T. ochraceipennis* from Chile and *T. pallidipennis* from Peru.

More than 50 species are currently recognized for the genus *Trimerotropis* in North America (Cigliano et al., 2017), including *T. cyaneipennis.* It is possible that *T. pallidipennis* could reach suitable sub-Andean areas in South America by the same events that allowed the expansion of other species into southern latitudes, giving rise to the Peruvian lineage of Lima. Nevertheless, one of the main features characterizing *T. cyaneipennis* (i.e., basal area of hind wing blue with a dark outer band) is absent in Clade II individuals, which in turn exhibit body shape and wing color that are similar to those of other *T. pallidipennis* specimens from South America. Therefore, the close relationship observed between Clade II and *T. cyaneipennis* deserves further investigation.

### Central Andes as source of endemism

The population analyses conducted on the *Trimerotropis pallidipennis* species complex from a very broad latitudinal and altitudinal range identified the Peruvian Andean region as an important hotspot for diversification. The level of differentiation among geographically close genetic lineages is higher in Peru than in other parts of its wide geographical distribution from North to South America. The results of our phylogenetic, biogeographic and molecular clock analyses allow us to propose a scenario for this incipient speciation process, in which both Pleistocene glaciations and asynchronous migrations of different stocks from North to South America would have caused secondary contacts between very distant genetic lineages in Peru.

Our biogeographical reconstruction suggests that the ancestor of the *T. pallidipennis* species group was probably distributed in North/Central America, as proposed by Confalonieri et al. (1998) and Husemann et al. (2013). After the closure of the Panama Isthmus (∼3 Mya, Fig. 2), and when climatic conditions turned the tropical environments of Central America into savanna or grassland type habitats (Raven, 1963; Woodburne, 2010; Wilson & Sipes, 2014), these arid-adapted *Trimerotropis* species were able to “cross the line”, reaching southern latitudes. The biogeographic analysis suggests that two different stocks -which had undergone allopatric differentiation from a common ancestor-, dispersed southward (∼1.3 Mya). This would have given rise to two lineages still coexisting in Peru: the coastal and the mountain lineages, which may correspond to those previously envisioned by Rehn (1939). Subsequent vicariant and dispersal events continued the process of differentiation, leading to the numerous genetic lineages (i.e., clades) detected here. Dating clearly indicates that many of these events occurred during the Late Pleistocene (Fig. 2), resulting in a hotspot of differentiation in the Peruvian Andes. At this time, the paleogeographic forces were relatively stable but paleoclimatic fluctuations led to the Pleistocene glaciations occurring in cycles of 100,000 years (Milankovitch’s cycles), characterized by an extensive development of substantial ice sheets (Ehlers & Gibbard, 2007). Therefore, the topography and the climatic conditions existing in the Peruvian highlands during the Pleistocene probably played a central role in the history of the populations studied here by preventing gene flow between them, which resulted in differentiation of many genetic lineages. Our research contributes with another case of “island diversification”, though we cannot assert if this process was strong or long enough to separate the various *Trimerotropis* lineages inhabiting the Peruvian Andes into different species.

The “island diversification” hypothesis is supported by the fact that other species would have experienced similar patterns of diversification in this same area (Chesser, 2000; Young & León, 2001; Hughes & Eastwood, 2006; Koscinski et al., 2008; Saez et al., 2014; Pocco et al., 2015). Thus, it is more probable that the same interplay of historical events affected several co-distributed taxa than that other demographic and/or ecological processes affected only one of them. For instance, a recent phylogenetic analysis of *Orotettix*, a flightless, brachypterous genus of grasshoppers composed of 10 species which occur in allopatry in transverse zones and deep valleys and/or in parapatry in Central Andes, also revealed a pattern of rapid diversification that probably took place during the Middle to Late Pleistocene (Pocco et al., 2015; MC Scattolini, VA Confalonieri, A Lira-Noriega, S Pietrokovsky, MM Cigliano, 2016–2017, unpublished data). Likewise, other grasshopper genera of Melanoplinae such as *Jivarus*, *Ponderacris, Tiyantiyana, Maeacris, Pediella* and *Huaylasacris* also constitute examples of endemism and recent species diversification in North and Central Andes (Cigliano & Amedegnato, 2010; Cigliano & Amedegnato, 2010; Cigliano, Pocco & Lange, 2011; Pocco et al., 2013).

In conclusion, our work illustrates the importance of phylogenetic and taxonomic studies to identify geographic areas as biodiversity hotspots. From an evolutionary approach, they increase our understanding of the interwoven processes which interacted in the past, giving rise to numerous new biological units distributed in a relatively restricted geographic area, like the Peruvian Andes. From a conservation approach, these studies provide valuable information that can be used by researchers, managers and policy makers engaged in biodiversity.

##  Supplemental Information

10.7717/peerj.3835/supp-1 Table S1 Sampling locations of species in the *Trimerotropis pallidipennis* complex The following data are provided: species names, specimen IDs, map codes corresponding to those in Fig. 1, collection locations and their coordinates, and GenBank accession numbers for each individual and gene. *T.* = Trimerotropis.

10.7717/peerj.3835/supp-2 Table S2 Comparison of results from biogeographic analyses

10.7717/peerj.3835/supp-3 Supplemental Information 1 The three aligments (COI, HIS and ITS) and the corresponding GenBank accession numbers

## Additional Information and Declarations

### Data Availability

The following information was supplied regarding data availability: GenBank: KX760166–KX760195, MF164183–MF164257.
