# Supplementary material for: Unraveling the diversification history of grasshoppers belonging to the “Trimerotropis pallidipennis” (Oedipodinae: Acrididae) species group: a hotspot of biodiversity in the Central Andes
Source: PeerJ. 2017 Sep 29;5:e3835. doi: 10.7717/peerj.3835 (PMC5624295; doi:10.7717/peerj.3835)
Supplement: Table S1 — The following data are provided: species names, specimen IDs, map codes corresponding to those in Fig. 1, collection locations and their coordinates, and GenBank accession numbers for each individual and gene. T. = Trimerotropis. [file peerj-05-3835-s001.doc]

**Supplementary Table S1**

**Table S1** Sampling locations of species in the *Trimerotropis pallidipennis* complex, with species names, specimen ID numbers, map codes corresponding to those in Figure 1, collection locations and their coordinates, and GenBank accession numbers for each individual and gene. *T.= Trimerotropis*

| Species | Map code | Country / State (location) | Coordinates | Samples | NCBI accession numbers | | | |
| --- | --- | --- | --- | --- | --- | --- | --- | --- |
|  |  |  |  |  | *ND5* | *COI* | ITS2 | *H3* |
| ***T. pallidipennis*** | **1** | **USA / Montana** | **N 45.43 / W 107.44** | **T105** | **JQ286671** | **JQ286539** | **JQ286718** | **JQ286598** |
|  | **2** | **USA / New Mexico** | **N 34.38 / W 106.44** | **T5** | **JQ286655** | **JQ286523** | **-** | **JQ286582** |
|  | **3** | **USA / Arizona** | **N 31.74 / W 109.95** | **T136** | **JQ286674** | **JQ286542** | **JQ286721** | **JQ286601** |
|  | **4** | **USA / Texas** | **N 38.86 / W 80.21** | **T85** | **JQ286673** | **JQ286541** | **JQ286720** | **JQ286600** |
|  | **5** | **Mexico / Salamanca** | **N 20.45 / W 101.18** | **T160** | **JQ286675** | **JQ286543** | **-** | **JQ286602** |
|  | **6** | **Mexico / Irapuato** | **N 20.40 / W 101.21** | **T140** | **JQ286665** | **JQ286533** | **-** | **JQ286592** |
| ***T. andeana*** | **7** | **Perú / Lima** | **S 12.035 / W 77.0186** | **96-1**  **96-2**  **96-4**  **96-5**  **96-6**  **96-7**  **96-8**  **96-9**  **96-10** | ******  ******  ******  ******  ******  ******  ******  ******  ****** | ******  ******  ******  ******  ******  ******  ******  ******  ****** | ******  ******  ******  ******  ******  ******  ******  ******  ****** | ******  ******  ******  ******  ******  ******  ******  ******  ****** |
|  |  |  |  |  |  |  |  |  |
|  | **8** | **Perú / Ancash** | **S 9.60 / W 77.51** | **P110** | **JQ286639** | **JQ286507** | ***** | **-** |
|  |  |  |  | **P318** | **JQ286693** | **-** | **JQ286742** | **-** |
|  |  |  |  |  |  |  |  |  |
|  |  |  |  | **P1028**  **P1029**  **P1030** | ******  **-**  **JQ286641** | **-**  **JQ286556**  **JQ286509** | **JQ286740**  **JQ286731**  **JQ286703** | **-**  **JQ286614**  **JQ286572** |
|  | **9** | **Perú / Cusco** | **S 14.02553 / W 71.26514** | **P25**  **P26** | **JQ286642**  **-** | **JQ286510**  **JQ286559** | **JQ286704**  **-** | **JQ286573**  **-** |
|  |  |  |  |  |  |  |  |  |
|  | **10** | **Perú / Pikillacta** | **S 13.6166 / W 71.7147** | **PK1**  **PK2**  **PK3**  **PK4** | ******  ******  ******  ****** | ******  ******  ******  ****** | ******  ******  ******  ****** | ******  ******  ******  ****** |
|  | **11**  **12** | **Perú / Puno**  **Peru/ Juliaca** | **S 16.0058 / W 69.7728**  **S 15.4908 / W 70.1269** | **77-2**  **P118** | ******  **-** | ******  **JQ286558** | ******  **-** | ******  **-** |
|  | **13** | **Perú / Arequipa** | **S 16.0744 / W 71.5074** | **78-1**  **78-2**  **78-3**  **78-4**  **78-5**  **78-6**  **78-7** | ******  ******  ******  ******  ******  ******  ****** | ******  ******  ******  ******  ******  ******  ****** | ******  ******  ******  ******  ******  ******  ****** | ******  ******  ******  ******  ******  ******  ****** |
| ***T. ochraceipennis*** | **13** | **Chile / Coquimbo** | **S 29.96 / W 71.33** | **T116**  **T117**  **T118**  **T119**  **T125**  **T126** | **JQ286681**  **JQ286679**  **JQ286678**  **JQ286680**  **JQ286683**  **JQ286682** | **JQ286549**  **JQ286547**  **JQ286546**  **JQ286548**  **JQ286551**  **JQ286550** | **JQ286725**  **JQ286723**  **JQ286722**  **JQ286724**  **JQ286726**  **-** | **JQ286607**  **JQ286605**  **JQ286604**  **JQ286606**  **JQ286609**  **JQ286608** |
| ***T. sp*** | **14** | **Argentina / Jujuy** | **S 22.887917 / W 65.249528** | **JU1**  **JU2** | **-**  ****** | **-**  ****** | **JQ286734**  ****** | **JQ286619**  ****** |
|  | **15** | **Argentina / Salta** | **S 25.1703 / W 65.1471** | **SA3** | ****** | ****** | ****** | ****** |
|  | **16** | **Argentina / Chaco** | **S26.9333 / W 61.5999** | **Cha3** | ****** | ****** | ****** | ****** |
|  | **17** | **Argentina / Córdoba** | **S 31.3732 / W 64.5232** | **C12C** | **-** | **-** | **-** | **JQ286618** |
|  | **18** | **Argentina / Mendoza** | **S 34.530 / W 68.350** | **G2** | **-** | **-** | **JQ286733** | **-** |
|  |  |  |  | **Me7** | ****** | ****** | ****** | ****** |
|  |  |  |  | **PI5** | ****** | ****** | ****** | ****** |
|  | **19** | **Argentina / San Luis** | **S 32.350 / W 69.200** | **US10** | ****** | ****** | ****** | ****** |
|  | **20** | **Argentina / Buenos Aires** | **S 38.490 / W 62. 420** | **Vi1** | **JQ286694** | **-** | **JQ286743** | **-** |
|  | **21** | **Argentina / Rio Negro** | **S 48.180 / W 71.475** | **CO3** | ****** | ****** | ****** | ****** |
| ***Outgroups*** |  |  |  |  |  |  |  |  |
| ***Sphingonotus corsicus*** | **-** | **France / Corse** | **N 41.400 / E 8.540** | **S1 (K263)** | **JQ286628** | **JQ286496** | **JQ286696** | **JQ286563** |
|  |  |  |  |  |  |  |  |  |
| ***T. maritima*** | **-** | **USA / Texas** | **N 31.5525 / W 97.2248** | **T1**  **T52** | **JQ286630**  **JQ286629** | **JQ286498**  **JQ286497** | **JQ286698**  **JQ286697** | **JQ286565**  **JQ286564** |
| ***Conozoa texana*** | **-** | **USA / New Mexico** | **N 34.4155 / W 106.4654** | **T15**  **T16** | **JQ286632**  **JQ286631** | **JQ286500**  **JQ286499** | **JQ286699**  **-** | **JQ286567**  **JQ286566** |
| ***T. saxatilis*** | **-** | **USA / Texas** | **N 33.18 / W 97.33** | **T132**  **T133** | **JQ286635**  **JQ286634** | **JQ286503**  **JQ286502** | **JQ286700**  ***** | **JQ286570**  **JQ286569** |
|  | **-** | **USA / Texas** | **N 30.96 / W 99.23** | **T174** | **JQ286633** | **JQ286501** | **-** | **JQ286568** |
|  |  |  |  |  |  |  |  |  |
| ***T. cyaneipennis*** | **-** | **USA/Arizona** | **N 31.74 / W 109.95** | **T3** | **KJ923364** | **KJ923416** | **-** | **KP201167** |
|  |  |  |  | **T4** | **KJ923365** | **KJ923417** | **-** | **KP201168** |
| ***T. latifasciata***  ***T. californica***  ***T. pistrinaria*** | **-** | **USA/ New Mexico**  **USA/ Montana**  **USA/ New Mexico**  **USA/ New Mexico** | **N 34.4155 / W 106.46**  **N 45.43 / W 107.44**  **N 34.4155 / W 106.46**  **N 34.4155 / W 106.46** | **T17**  **T18**  **T110**  **T28**  **T31** | **KJ923363**  **JQ513040**  **JQ513163**  **JQ513160**  **JQ513165** | **KJ923415**  **JQ513161**  **JQ513047**  **JQ513048**  **JQ513046** | **-**  **-**  **JQ513119**  **JQ513116**  **JQ513121** | **KP201166**  **JQ513206**  **JQ513208**  **JQ513205**  **JQ513210** |

*: ITS2 sequences used in this study, but with less than 200 bp. Not included in Genbank.

** NCBI accession numbers will be released upon publication.
