# Supplementary material for: Unraveling the diversification history of grasshoppers belonging to the “Trimerotropis pallidipennis” (Oedipodinae: Acrididae) species group: a hotspot of biodiversity in the Central Andes
Source: PeerJ. 2017 Sep 29;5:e3835. doi: 10.7717/peerj.3835 (PMC5624295; doi:10.7717/peerj.3835)
Supplement: Table S2 [file peerj-05-3835-s002.docx]

**Supplementary Table S2**

**Table S2**: Comparison of results derived from biogeographic analyses. S-DIVA: Statistical dispersal-vicariance analysis, DEC: analysis dispersal-extinction-cladogenesis analysis and S-DEC: statistical dispersal-extinction-cladogenesis analysis using the program RASP v 3.2. Relative probabilities (RP) of the ancestral areas are given as a fraction of the global likelihood of a split (MA: indicates those ancestral areas with low RP). Most likely events route for each node are provided. The areas are coded following those in Figure 2.

|  | **S-DIVA** | |  | **DEC** |  |  | **S-DEC** | |  |
| --- | --- | --- | --- | --- | --- | --- | --- | --- | --- |
|  | AA | RASP ROUTE | RP | AA | RASP ROUTE | RP | AA | RASP ROUTE | RP |
| **Node 1** | B | B->B^B->B\|B | 100 | B | B->B^B->B\|B | 71.96 | B | B->B^B->B\|B | 68.7 |
|  |  |  |  | BD |  | 28.04 | BD |  | 30.69 |
|  |  |  |  |  |  |  | AB |  | 0.62 |
| **Node 2** | B | B->B^B->B\|B | 100 | B | B->B^B->BD^B->BD\|B | 51.95 | B | B->B^B->BD^B->BD\|B | 54.46 |
|  |  |  |  | BD |  | 37.6 | BD |  | 43.55 |
|  |  |  |  | AB |  | 10.45 | AB |  | 1.98 |
| **Node 3** | B | B->B^B^D->BD^B^D->BD\|BD Dispersal :2 | 70.71 | BD | BD->BD^B^D->BD\|BD Dispersal :2 | 60.35 | BD | BD->BD^B^D->BD\|BD Dispersal :2 | 70.66 |
|  | BD |  | 22.56 | B |  | 22.76 | B |  | 24.68 |
|  | MA |  | <4 | AB |  | 16.89 | AB |  | 4.66 |
| **Node 4** | BD | BD->B\|D Vicariance:1 | 100 | BD | BD->B\|D Vicariance:1 | 100 | BD | BD->B\|D Vicariance:1 | 97.88 |
|  |  |  |  |  |  |  | MA |  | <2 |
| **Node 5** | BD | BD->BAD->B\|AD Dispersal :1 Vicariance:1 | 42.64 | BD | BD->BAD->B\|AD Dispersal :1 Vicariance:1 | 62.86 | BD | BD->BAD->B\|AD Dispersal :1 Vicariance:1 | 76.91 |
|  | ABD |  | 28.68 | AB |  | 37.14 | AB |  | 21.25 |
|  | AB |  | 28.68 |  |  |  | MA |  | <2 |
| **Node 6** | AD | AD->D\|A Vicariance:1 | 100 | AD | AD->D\|A Vicariance:1 | 100 | AD | AD->D\|A Vicariance:1 | 90.81 |
|  |  |  |  |  |  |  | D |  | 9.2 |
| **Node 7** | D | D->D^D->D\|D | 100 | D | D->D^D->D\|D | 100 | D | D->D^D->D\|D | 99.02 |
|  |  |  |  |  |  |  | CD |  | 0.98 |
| **Node 8** | D | D->D^D->D\|D | 94.85 | D | D->D^D->D\|D | 100 | D | D->D^D->D\|D | 90.69 |
|  | CD |  | 21.6 |  |  |  | CD |  | 9.31 |
| **Node 9** | A | A->A^A->A\|A | 100 | A | A->A^A->A\|A | 100 | A | A->A^A->A\|A | 100 |
| **Node 10** | D | D->D^D->D\|D | 100 | D | D->D^D->D\|D | 100 | D | D->D^D->D\|D | 100 |
| **Node 11** | D | D->D^D->DC^D->D\|CD Dispersal :1 | 76.54 | D | D->D^D->DC^D->D\|CD Dispersal :1 | 100 | D | D->D^D->DC^D->D\|CD Dispersal :1 | 67.79 |
|  | CD |  | 23.46 |  |  |  | CD |  | 32.21 |
| **Node 12** | D | D->D^D->D\|D | 100 | D | D->D^D->D\|D | 100 | D | D->D^D->D\|D | 100 |
| **Node 13** | B | B->B^B->B\|B | 100 | B | B->B^B->B\|B | 100 | B | B->B^B->B\|B | 100 |
| **Node 14** | D | D->D^D->D\|D | 100 | D | D->D^D->D\|D | 100 | D | D->D^D->D\|D | 100 |
| **Node 15** | B | B->B^B->B\|B | 100 | B | B->B^B->B\|B | 100 | B | B->B^B->B\|B | 100 |
| **Node 16** | CD | CD->D\|C Vicariance:1 | 100 | CD | CD->D\|C Vicariance:1 | 100 | CD | CD->D\|C Vicariance:1 | 78.72 |
|  |  |  |  |  |  |  | D |  | 21.28 |
| **Node 17** | D | D->D^D->D\|D | 100 | D | D->D^D->D\|D | 100 | D | D->D^D->D\|D | 100 |
| **Node 18** | C | C->C^C->C\|C | 100 | C | C->C^C->C\|C | 100 | C | C->C^C->C\|C | 100 |
| **Node 19** | D | D->D^D->D\|D | 100 | D | D->D^D->D\|D | 100 | D | D->D^D->D\|D | 100 |
