# Supplementary material for: Unraveling the diversification history of grasshoppers belonging to the “Trimerotropis pallidipennis” (Oedipodinae: Acrididae) species group: a hotspot of biodiversity in the Central Andes
Source: PeerJ. 2017 Sep 29;5:e3835. doi: 10.7717/peerj.3835 (PMC5624295; doi:10.7717/peerj.3835)
Supplement: Supplemental Information 1 [file peerj-05-3835-s003.zip › GenBank sequence numbers.rtf]

COI_sequin_23_05_17  P3_18     MF164183COI_sequin_23_05_17  P10_28    MF164184COI_sequin_23_05_17  US10      MF164185COI_sequin_23_05_17  CO3       MF164186COI_sequin_23_05_17  PI5       MF164187COI_sequin_23_05_17  Me7       MF164188COI_sequin_23_05_17  Vi1       MF164189COI_sequin_23_05_17  Cha3      MF164190COI_sequin_23_05_17  C12C      MF164191COI_sequin_23_05_17  SA3       MF164192COI_sequin_23_05_17  JU1       MF164193COI_sequin_23_05_17  JU2       MF164194COI_sequin_23_05_17  G2        MF164195COI_sequin_23_05_17  PK1       MF164196COI_sequin_23_05_17  PK2       MF164197COI_sequin_23_05_17  PK3       MF164198COI_sequin_23_05_17  PK4       MF164199COI_sequin_23_05_17  78_1      MF164200COI_sequin_23_05_17  78_2      MF164201COI_sequin_23_05_17  78_3      MF164202COI_sequin_23_05_17  78_4      MF164203COI_sequin_23_05_17  78_5      MF164204COI_sequin_23_05_17  78_6      MF164205COI_sequin_23_05_17  78_7      MF164206COI_sequin_23_05_17  96_2      MF164207COI_sequin_23_05_17  96_5      MF164208COI_sequin_23_05_17  96_6      MF164209COI_sequin_23_05_17  96_7      MF164210COI_sequin_23_05_17  96_8      MF164211COI_sequin_23_05_17  96_9      MF164212COI_sequin_23_05_17  96_10     MF164213COI_sequin_23_05_17  96_4      MF164214COI_sequin_23_05_17  96_1      MF164215COI_sequin_23_05_17  77_2      MF164216H3_sequin_26_05_17   P3_18     MF164217H3_sequin_26_05_17   PI4       MF164218H3_sequin_26_05_17   Me5       MF164219H3_sequin_26_05_17   CA7       MF164220H3_sequin_26_05_17   CA1       MF164221H3_sequin_26_05_17   77_1      MF164222H3_sequin_26_05_17   77_2      MF164223H3_sequin_26_05_17   78_1      MF164224H3_sequin_26_05_17   78_2      MF164225H3_sequin_26_05_17   78_3      MF164226H3_sequin_26_05_17   78_4      MF164227H3_sequin_26_05_17   78_5      MF164228H3_sequin_26_05_17   78_6      MF164229H3_sequin_26_05_17   78_7      MF164230H3_sequin_26_05_17   PK1       MF164231H3_sequin_26_05_17   PK3       MF164232H3_sequin_26_05_17   96_1      MF164233H3_sequin_26_05_17   96_2      MF164234H3_sequin_26_05_17   96_3      MF164235H3_sequin_26_05_17   96_4      MF164236H3_sequin_26_05_17   96_5      MF164237H3_sequin_26_05_17   96_6      MF164238H3_sequin_26_05_17   96_7      MF164239H3_sequin_26_05_17   96_8      MF164240H3_sequin_26_05_17   96_9      MF164241H3_sequin_26_05_17   96_10     MF164242ITS2_sequin_26_05_17 96_4      MF164243ITS2_sequin_26_05_17 96_2      MF164244ITS2_sequin_26_05_17 96_6      MF164245ITS2_sequin_26_05_17 96_9      MF164246ITS2_sequin_26_05_17 77_1      MF164247ITS2_sequin_26_05_17 77_2      MF164248ITS2_sequin_26_05_17 78_2      MF164249ITS2_sequin_26_05_17 PK1       MF164250ITS2_sequin_26_05_17 PK2       MF164251ITS2_sequin_26_05_17 PK4       MF164252ITS2_sequin_26_05_17 SA1       MF164253ITS2_sequin_26_05_17 CO9C      MF164254ITS2_sequin_26_05_17 JU2       MF164255ITS2_sequin_26_05_17 Vi3       MF164256ITS2_sequin_26_05_17 JU1       MF164257
